# Clinical Course, Neurobiology and Therapeutic Approaches to Treatment Resistant Schizophrenia. Toward an Integrated View

**DOI:** 10.3389/fpsyt.2019.00601

**Published:** 2019-09-03

**Authors:** Cheryl Cheuk-Yan Leung, Romayne Gadelrab, Chukwuma Uchenna Ntephe, Philip K. McGuire, Arsime Demjaha

**Affiliations:** ^1^Department of Old Age Psychiatry, Institute of Psychiatry, Psychology, and Neuroscience (IoPPN), King’s College London, London, United Kingdom; ^2^South London and Maudsley NHS Foundation Trust, London, United Kingdom; ^3^Department of Psychosis Studies, Institute of Psychiatry, Psychology, and Neuroscience (IoPPN), King’s College London, London, United Kingdom; ^4^National Institute for Health Research (NIHR) Biomedical Research Centre (BRC), South London and Maudsley NHS Foundation Trust, London, United Kingdom

**Keywords:** schizophrenia, treatment-resistant, neurobiology, neuroimaging, clozapine

## Abstract

Despite considerable psychotherapeutic advancement since the discovery of chlorpromazine, almost one third of patients with schizophrenia remain resistant to dopamine-blocking antipsychotics, and continue to be exposed to unwanted and often disabling side effects, but little if any clinical benefit. Even clozapine, the superior antipsychotic treatment, is ineffective in approximately half of these patients. Thus treatment resistant schizophrenia (TRS), continues to present a major therapeutic challenge to psychiatry. The main impediment to finding novel treatments is the lack of understanding of precise molecular mechanisms leading to TRS. Not only has the neurobiology been enigmatic for decades, but accurate and early detection of patients who are at risk of not responding to dopaminergic blockade remains elusive. Fortunately, recent work has started to unravel some of the neurobiological mechanisms underlying treatment resistance, providing long awaited answers, at least to some extent. Here we focus on the scientific advances in the field, from the clinical course of TRS to neurobiology and available treatment options. We specifically emphasize emerging evidence from TRS imaging and genetic literature that implicates dysregulation in several neurotransmitters, particularly dopamine and glutamate, and in addition genetic and neural alterations that concertedly may lead to the formation of TRS. Finally, we integrate available findings into a putative model of TRS, which may provide a platform for future studies in a bid to open the avenues for subsequent development of effective therapeutics.

## Introduction

Almost one third of patients with schizophrenia do not respond to dopamine (DA) blocking antipsychotic medication and are described as being treatment-resistant (1). Although clozapine can be effective in these patients, there is usually a long delay before it is used, and what is more around half of treatment-resistant patients do not respond to clozapine (2, 3). Treatment-resistant schizophrenia (TRS), is thus associated with particularly poor clinical outcomes (4), and presents a major therapeutic challenge to psychiatry. One of the main impediments to finding novel treatments for TRS patients is the lack of understanding of the molecular basis of TRS, despite over 50 years of scientific work in this field. Moreover, biomarkers that can identify patients who are unlikely to respond to conventional treatment remain elusive. Fortunately, recent work has started to unravel some of the mechanisms underlying treatment resistance. Here we describe these scientific advances and propose an integrated model of TRS that may facilitate the identification of biomarkers for TRS and provide a rationale for the development of novel therapeutic approaches.

### Defining Treatment-Resistant Schizophrenia

Prior to embarking on finding reliable biomarkers and conduct promising clinical trials, it is of crucial importance to precisely stratify patients according to their response to treatment. The literature, however, has been limited by inconsistent TRS definitions. In the absence of a universally accepted definition, studies have opted for different TRS criteria according to their aims and population studied. This has resulted in marked heterogeneity in results and disparity in response rates. For instance, in Suzuki and colleagues’ systematic review, 33 studies reported treatment response rates ranging from 0% to 76% (5). Studies recruiting patients for novel antipsychotic drug trials may use more stringent criteria than those testing psychosocial interventions, thus reporting lower prevalence of TRS (5, 6).

Furthermore, the lack of precise and universal operational definitions of TRS may have important clinical and scientific implications. For instance, it hinders early detection of treatment resistance and, subsequently, may delay initiation of clozapine, and in research settings, it complicates comparisons and interpretation of results. To address these issues, International Treatment Response and Resistance in Psychosis (TRRIP) group has developed operationalized TRS definition criteria and reached consensus on “minimum requirements.” The group emphasizes that any definition of treatment resistance should indicate that the patient has received an adequate trial of antipsychotic medication in terms of dosage (equivalent to or greater than 600 mg of chlorpromazine per day), trial of two different antipsychotics for a duration of 6 weeks each at a therapeutic dose, strong advocation for acquiring treatment adherence measures (≥80% of prescribed doses), as well as the use of structured clinical assessments to ascertain symptom presence and severity (7). However, there are limitations to these criteria, such as the use of dichotomous classification, which does not account for the continuum of treatment response. As authors acknowledge, future revisions incorporating novel neurobiological findings are required prior to criteria being fully standardized and more applicable across research and clinical settings.

## Heterogeneity of Clinical Course of TRS

For decades, researchers in the field of TRS debated whether treatment resistance is a stable phenotype, or whether it is a consequence of neurodegenerative process, evolving over time in the context of multiple episodes and repeated exposure to antipsychotic treatment.

In favor of the first notion were reports that emerged even prior to the existence of psychopharmacology, and indirectly suggested that unfavorable clinical outcomes may be related to more severe and enduring subtype of schizophrenic illness. Kraepelin, referring in his textbook to Albrecht’s observations that one third of his cases with hebephrenia reached terminal state within a year of onset, concluded: “Often enough the unmistakable symptoms of dementia appear already within the first year” (8). Decades later, Kolakowska and colleagues demonstrated that the majority of “poor responders” were unresponsive throughout their illness and remarked that treatment resistance was related to the “type,” rather than the “stage” of schizophrenia. (9). Analogously, two prospective studies observed that resistance to treatment was apparent in early stages of illness (10, 11). Furthermore, longitudinal first episode psychosis (FEP) studies (12) observed that between 5% and 25% of patients were unresponsive and had persistent positive symptoms during the initial phase of illness (12, 13). Such variability again, might be a consequence of the different TRS criteria employed in these studies.

Other authors, however, considered neurodegenerative hypothesis, attributing treatment resistance to chronicity of illness. Wyatt (1991) reviewed the evidence derived from 22 studies of predominantly FEP patients and concluded that early psychopharmacological intervention could improve the outcomes and prognosis of the disorder (14). It was proposed that a neurodegenerative process might be inherent to psychosis and thus adversely affect the clinical course in those who were non-compliant with treatment and subjected to multiple relapses.

More recently, the largest to date, a 10-year follow-up FEP study, designed to address these inconsistencies in literature, found that over 80% of treatment-resistant patients were persistently resistant from the very early stage of their illness (15).

This work, however, identified a small proportion of patients (16%), who although initially responded to medication, ultimately developed treatment resistance. These patients showed higher number of relapses associated with more inpatient admissions. The reasons for this remain elusive and warrant further exploration. As suggested by animal studies, it can be that chronic treatment with DA blocking agents may induce D2 receptor up-regulation leading to breakthrough DA supersensitivity, which may predispose some patients to treatment resistance (16, 17). Accordingly, it has been shown that in a proportion of patients not only the time to remission is longer in subsequent episodes, but less, if at all, achievable (18, 19). Furthermore, most recently, a study by Takeuchi et al. (20) has implicated that treatment response is unfavorably affected by symptomatic relapse following initial response. This finding could be particularly relevant to this subgroup of patients (20).

**Figure 1:**
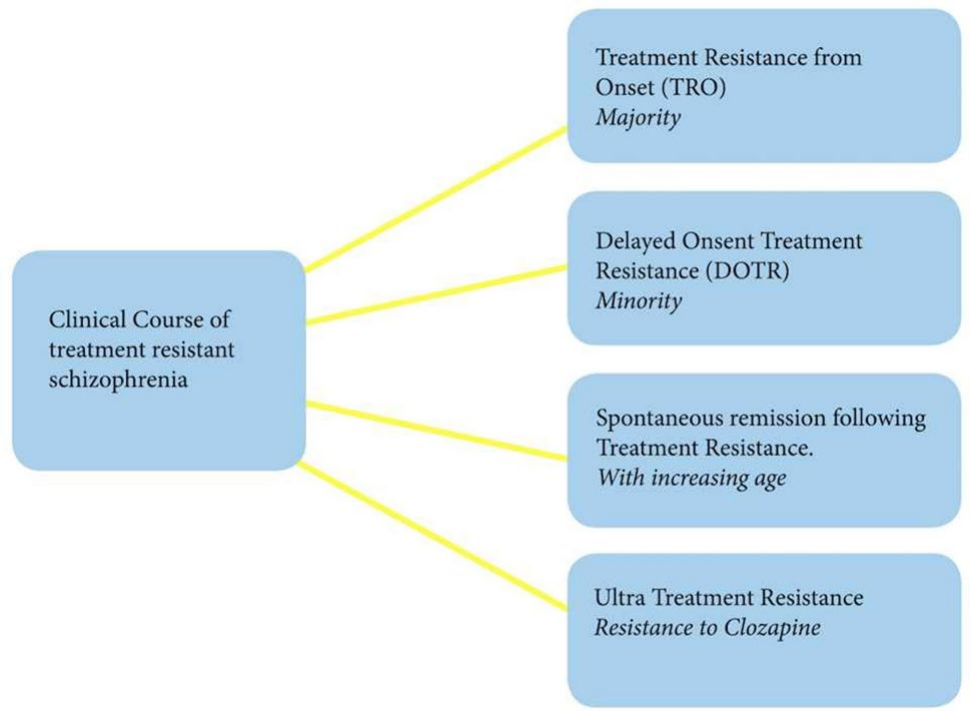
Clinical course of treatment-resistant schizophrenia.

On the other hand, some treatment-resistant patients may achieve spontaneous remission or begin responding to treatment later in life (21), which is in line with previous observations that older patients with schizophrenia require much less intensive maintenance antipsychotic treatment than those who are younger in age (22–24). This can perhaps be explained by the fact that DA system is age-dependent, with significant reductions in dopaminergic transmission in older patients being observed (24, 25). This notion is intriguing and contradicts the recent findings of unaltered DA levels in TRS, but it can be that this sub-group of patients have different neurobiology altogether, which remains to be determined in larger and more stratified studies. Finally, up to 50% of treatment-resistant patients are resistant to clozapine recently termed as “ultra-treatment resistance” (26). Such non-response to clozapine, a last treatment resort for those who do not respond to first-line antipsychotics, is the major unmet clinical need in schizophrenia (**Figure 1**).

### Putative Predictors of TRS

Several studies have identified younger age at onset, longer duration of untreated psychosis, and negative symptomatology to be associated with treatment resistance (12, 15, 27, 28). Furthermore, severe cognitive impairment, poorer premorbid functioning (21, 29), obstetric complications (30), as well as neurological soft signs (31), have, in addition, shown significant associations with treatment resistance. Additionally, family history and, thus, increased genetic burden (32, 33) have been linked to poor prognosis of illness, and finally, a study comparing first-degree relatives of patients with and without TRS showed higher morbidity risk of schizophrenia in relatives of TRS patients (34).

The findings and observations, to date, indicate that treatment resistance in schizophrenia is heterogenous, as a disorder itself, assuming at least four different trajectories. However, a significant majority of patients with TRS appear to be resistant at the time of their first presentation. This form of treatment resistance may represent an enduring phenotype of schizophrenic illness, which is particularly associated with younger age at onset and negative symptoms (15). Such high prevalence at the early phase of illness should alert clinicians to commence clozapine as soon as possible. However, larger FEP studies are needed to delineate reliable predictors to facilitate early and accurate detection of patients who are not likely to respond to first-line antipsychotic treatment.

## Neurobiology of Treatment-Resistant Schizophrenia

Until recently, the underlying neurobiology of treatment-resistant schizophrenia remained elusive. Emerging evidence from TRS imaging and genetic literature implicates dysregulation in several neurotransmitters, particularly DA and glutamate, and in addition genetic and neural alterations that concertedly may lead to the formation of treatment resistance in schizophrenia. The presented literature here is not exhaustive. Instead, we predominantly focus on the most robust and high-impact evidence and neurobiological aspects that may predispose to treatment resistance.

### Neurotransmitters in TRS

The DA hypothesis remains an important part of our understanding of psychosis. DA blocking antipsychotics are effective in a majority of patients with schizophrenia, and illicit drugs that induce acute psychotic symptoms increase DA levels. Although this hypothesis does hold true for the patients who are responsive to treatment, it fails to provide explanations for patients with TRS.

To understand the dopaminergic mechanisms underlying treatment resistance, scientists have first focused on striatal DA D2 receptors. Positron emission tomography (PET) studies revealed significant associations between striatal DA D2 occupancy and prediction of short-term clinical response to antipsychotic treatment (35) and suggested that at least 60% of DA D2 receptor occupancy is necessary to reach adequate therapeutic response. This is true for both typical and atypical antipsychotics (31, 35, 36), excluding clozapine (37). Hypothesizing that the lack of response may result from inadequate DA D2 receptor blockade, Wolkin et al. (38), using PET, examined DA D2 receptor occupancy in patients with TRS schizophrenia and intriguingly demonstrated almost identical striatal DA D2 receptor occupancies in both treatment-responsive and treatment-resistant patients (38). Correspondingly, a [123I] IBZM Single Photon Emission Tomography (SPET) study reported a similar degree of DA D2 occupancy in both groups (39). Moreover, Kane et al. (40) in their seminal trial included the most severely ill and treatment-resistant patients with schizophrenia who failed a prospective trial of haloperidol at doses of at least 60 mg/day, which indirectly suggests that DA receptor occupancy was sufficiently achieved (40).

**Figure 2:**
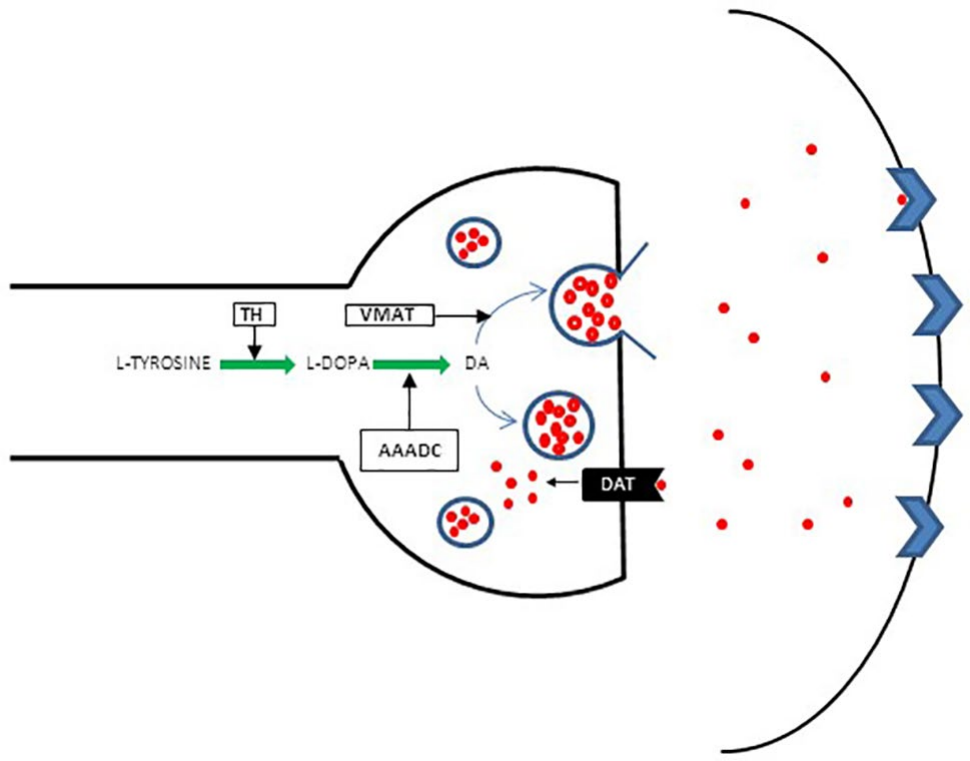
Schematic presentation of presynaptic DA regulation. Conversion of l-tyrosine (4-hydroxyphenylalanin) to l-3, 4 dihydroxyphenylalanine [l-DOPA] constitutes the first step in a complex pathway of DA synthesis. l-tyrosine is derived mainly from dietary sources, although a small quantity originates from l-phenylalanine converted to l-tyrosine by phenylalanine hydroxylase (PHA). l-tyrosine is converted to l-DOPA by tyrosine hydroxylase (TH). Aromatic L-amino acid decarboxylase (AAADC) then acts on l-DOPA to convert it to DA. The DA uptake transporter (DAT) plays an additional role in increasing cytoplasmic DA levels via the reuptake of extracellular DA and thus maintains extracellular DA homeostasis. From the cytoplasm, the majority of DA is stored in specialized synaptic vesicles by the vesicular monoamine transporter (VMAT) and is ready for release upon arrival of the action potential.

It became apparent that although DA D2 blockade may be necessary, it does not guarantee a response. Thus, the focus shifted to investigating presynaptic DA synthesis capacity (DSC). Increased DSC in patients with schizophrenia is considered one of the most replicated finding in dopaminergic studies of schizophrenia (41–43), and therefore, DSC anomalies are considered critical in the formation of positive psychotic symptoms. The biochemistry of DA synthesis is presented in a schematic diagram (**Figure 2**).

The first study to directly examine DSC in specifically treatment-resistant patients with schizophrenia, using PET and stringent criteria for TRS, demonstrated unaltered DSC in treatment-resistant patients. In a subgroup of these patients, authors measured glutamate levels using proton magnetic resonance spectroscopy (^1^H-MRS) and documented increased glutamatergic levels in anterior cingulate cortex (ACC) in TRS patients (44). Analogously, two subsequent studies reported increased glutamate levels in the ACC in treatment-resistant patients, but decreased levels in treatment responders (45, 46). To address the potential effects of medication or chronicity on these findings, the same group prospectively investigated DSC and ACC glutamate levels in initially medication-naïve FEP patients and confirmed that although striatal DSC is unaltered, ACC glutamate levels are increased in patients who subsequently do not respond to treatment (47). Most recently, in a multicenter longitudinal study of either minimally treated or medication naïve patients, higher levels of glutamate in the ACC were associated with treatment non-response to amisulpiride (48).

However, not all studies have observed glutamatergic alterations in relation to treatment response as discussed in recent systematic reviews (49, 50). The discrepancy in results may be related to differing methodology and, in particular, different TRS criteria studies. Thus, some studies may have misclassified TRS patients as responders or vice versa, which may lead to different outcomes and complicate comparisons as we discussed in previous sections.

Taken together, the neurochemical evidence to date supports the hypothesis that distinct neurochemical abnormalities, such as normal striatal DSC and increased ACC glutamate function, may underlie TRS. What is more, the demonstrated lack of DA abnormality in this subgroup of patients raises the possibility that other neurotransmitters, such as GABAaergic, glutamatergic, and endocannabinoid systems may be a promising target for novel antipsychotics.

However, the neurobiological underpinning of schizophrenia in general as well as that of TRS may involve complex interactions of these neurotransmitters. Carlsson and colleagues (2000, 2001) proposed that alterations in cortical glutamate levels, either acting directly as an “accelerator” or *via* GABA interneuron projections as a “brake,” modulates the firing of dopaminergic neurons that can in turn lead to either decrease or increase in dopaminergic activity (51, 52). Thus, for instance, the reduced glutamate activity enhances DA release in dopaminergic pathways, which then *via* negative feedback, mediated, at least in part, *via* the striatum and the thalamus, regulates glutamate release that would then act as “a brake” on cortical DA production (51, 52). How this mechanism operates in TRS remains to be determined in precise future pre-clinical models. At this stage, and based on available neurochemical imaging evidence, we could only speculate that in TRS, this mechanism involves the indirect pathway that involves GABA interneurons that exerts a “brake” effect on DA production, which may explain the absence of DSC increase in TRS. In turn, the absence of feedback from normal striatal DA status may lead to cortical hyperglutamatergia. In line with this, studies have reported an inverse correlation between cortical glutamate and striatal DSC (53, 54).

Genetic data also support to some degree the distinct neurobiology of TRS by suggesting a specific heritable vulnerability in TRS sub-group of patients. It has been suggested that TRS may be related to increased genetic burden (32). For instance, family history of psychosis has been shown to be associated with TRS (33). Studies that investigated several candidate genes, such as ABCB1, ABCC1, and ABCB11, demonstrated associations with response to antipsychotics as summarized by Vita et al. (50). Subsequent studies have examined polygenic risk scores (PRS) representing aggregate score of risk loci, that have been identified from genome-wide association studies (GWAS) in schizophrenia patients, to determine whether this approach can detect treatment non-response, but both chronic and medication-naive FEP studies have been negative (55, 56).

### Functional and Structural Neuroimaging

Evidence from structural magnetic resonance imaging (MRI) studies indicates that patients with limited response to treatment have increased cortical atrophy in comparison with responders (57, 58). Reduced gyrification was observed across multiple brain regions at illness onset in FEP patients who subsequently do not respond to treatment (59). In addition, cortical thinning generally, but particularly in dorsolateral prefrontal cortex (DLPFC) was reported in TRS (60). Recent review has revealed that patients with TRS have larger number of regions with decreased GM when compared with responders (49).

Functional MRI studies have similarly been able to distinguish between responders and non-responders. Most recently, global functional connectivity decrease, particularly in frontotemporal and occipital regions, was reported to be associated with treatment resistance in several studies (61, 62). Two comprehensive reviews have demonstrated decreased metabolism in the prefrontal and frontotemporal regions and hypermetabolism in the basal ganglia in TRS patients (63, 64).

## Therapeutic Approaches of Treatment-Resistant Schizophrenia

### Clozapine—A Gold Standard

The discovery of chlorpromazine has stimulated the discovery of numerous DA-blocking antipsychotics that in most patients are effective. However, first-line antipsychotic treatment in considerable proportion of patients does not alleviate symptoms, but instead exposes these patients to unwanted and often disabling side effects. The only antipsychotic, to date, that has an adequate therapeutic effect in this subgroup of patients is clozapine, and as such remains superior to other antipsychotics for TRS patients (65–67).

Clozapine has been actualized by Kane and colleagues in their seminal work (40). They have shown clozapine to be more effective than chlorpromazine at symptomatic reduction (30% vs. 4%, respectively) in participants who failed a trial of haloperidol treatment (40). It is, however, underutilized (68) with documented delay of its initiation approximating 5 years (69). This delay has important clinical implications associated with reduced effectiveness, increased number of hospital admissions, and more frequent use of concurrent electroconvulsive therapy (ECT) (68, 70–72). Recent scientific reports advocate its use at much earlier stages of illness (15, 73). This is compounded by the fact that a great majority of TRS patients seem to be destined to non-response to medication at the earliest stages of their illness necessitating much earlier use of clozapine (15). A meta-analysis by Okhuijsen-Pfeifer and colleagues (2018) comparing clozapine with a number of conventional antipsychotics found significant benefit for early clozapine use (Hedges’ *g* = 0.220; *P* = 0.026; 95% CI = 0.026–0.414) (74), whereas a large three-phase switching clinical trial conducted by the OPTiMiSe study group found that following a failed initial response to amisulpiride switching to olanzapine resulted in no additional benefit, whereas switching to clozapine did improve clinical outcomes (73).

The precise psychopharmacology of clozapine is yet to be unraveled. Its efficacy in TRS may be related to the fact that clozapine is a weak DA blocker and that its action may be mediated *via* glutamatergic and serotonergic pathway as indicated by recent neurochemical imaging literature (45, 47, 75–79).

## Treatment Strategies in Ultra-Treatment Resistance

### Clozapine Augmentation With Other Psychotropic Agents

Almost half of TRS patients do not respond to clozapine (2, 3, 40, 80) and are termed ultra-treatment resistant. When faced with such treatment challenge, clinicians tend to resort to augmentation with other psychotropic agents, although there is limited evidence to support this therapeutic approach (32, 81). Antipsychotics are the most frequently utilized and studied agent, and of these, risperidone is the most frequently researched (82). A meta-analysis has shown no increased benefit to augmentation with risperidone (83) and another of 14-placebo controlled RCTs showed that augmentation with antipsychotic medication is of little benefit (effect size, −0.239; 95% CI, −0.45 to −0.026; *P* = 0.028) (84). Furthermore, augmentation with antipsychotics seems to be associated with a worsening of side effects (83). Similarly, the augmentation with mood stabilizers and SSRIs has yielded limited evidence for efficacy (85). Lamotrigine has garnered conflicting evidence (83, 86). While topiramate has some evidence supporting its effect at curtailing weight gain in patients taking clozapine, there is limited evidence for its reduction in psychotic symptoms (85). Augmentation, in theory, may be a useful approach to adopt in managing TRS as it utilizes already existing medication, whose mechanisms of action and side effect profiles have been well studied. Unfortunately, the evidence does not currently support their effectiveness.

### Other Treatment Strategies

Evidence investigating the effectiveness of ECT in combination with clozapine has shown positive results (87). A recent meta-analysis by Wang and colleagues (2018) who analyzed data from 18 randomized control trials (n = 1769) found that adjunctive ECT was more beneficial for short-term recovery, compared with clozapine alone (standard mean difference = −0.54; 95% CI, −0.88 to −0.20; I2 = 77%, *P* = 0.002) (88).

Repetitive trans-magnetic stimulation (89) and transcranial direct stimulation (90) may be effective at reducing auditory hallucinations, though some of their effects may be short-lived (91). Their low-cost and mild side effect profile (92, 93) make them attractive options to treat schizophrenia and, more specifically, TRS, though with a scarcity of large clinical trials, more research is needed to delineate their effectiveness as sole or adjunct agents (81).

In summary, clozapine remains a gold standard treatment for patients with TRS (74). Research has shown that delay in clozapine initiation leads to a poorer response to treatment (72) and, worse outcomes (70, 71). However, there are significant issues with its tolerability, and there is still a significant subgroup of non-responders to clozapine who see, a modest, if any improvement with pharmacological (32, 81) and non-pharmacological augmentation (87, 89, 90). This strongly supports a need for new therapeutic targets. Recent meta-analytic work has demonstrated significant effects of glutamatergic agents, such as glycine/d-serine site antagonists, on negative symptoms (94) that are generally resistant to DA-blocking antipsychotics. In view of complex interplay of neurotransmitters governing schizophrenia and particularly treatment resistance, other promising therapeutic approaches include the stimulation of GABA receptors to overcome glutamatergic deficits, which is yet to be tested in clinical trials (95), as well as the use of cannabinoids, which have shown promising therapeutic effect in recent drug trials (96–98).

## Conclusion

**Figure 3:**
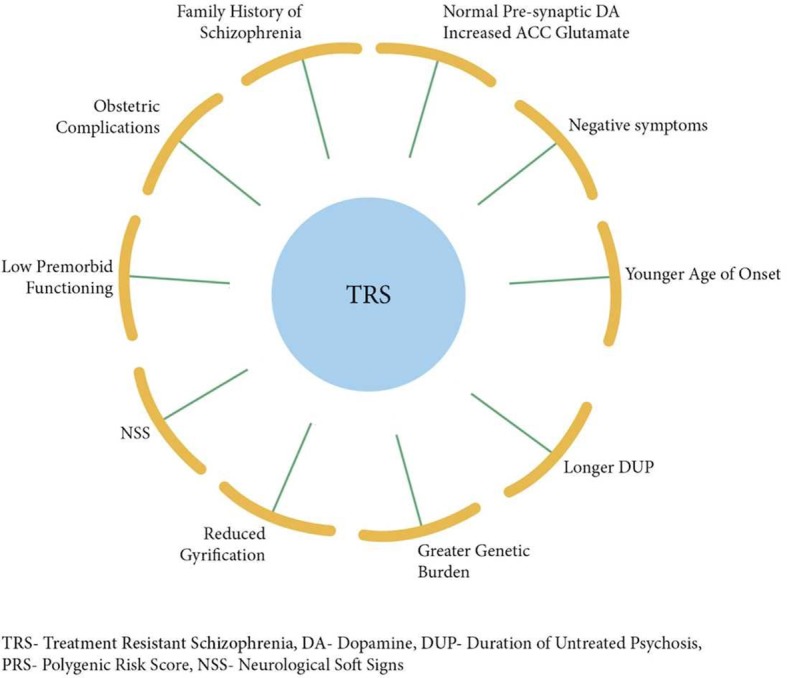
Putative model integrating factors that are associated with treatment resistance in schizophrenia.

Taken together, the findings to date suggest that TRS is a distinct, more severe, and enduring subtype of schizophrenic illness, marked by greater neuroanatomical abnormalities and different molecular mechanisms. Such complex and intractable condition requires a more fine-grained conceptualization of underlying neurobiology, which may consequently lead to much-needed novel biologically determined treatments. It is crucial to develop clinical tools that will enable clinicians to predict whether a patient will or will not respond to DA blockade, so that clozapine or other novel alternatives can be commenced as early as possible. Here, we integrate the available findings into a putative predictive model of TRS (**Figure 3**), which may provide a platform for impending scientific developments. Carefully designed studies that address rigorously the heterogeneity of the disorder and that of the antipsychotic treatment response (97, 99) are urgently needed so that patients may be stratified accurately according to their likely therapeutic responses.

## Author Contributions

All authors contributed significantly to the conception and drafting of the manuscript. AD critically reviewed the manuscript and all authors approved the final version of the manuscript.

## Conflict of Interest Statement

The authors declare that the research was conducted in the absence of any commercial or financial relationships that could be construed as a potential conflict of interest.
